# Focus group in the development of concepts for a Nursing model: experience report

**DOI:** 10.1590/0034-7167-2022-0689

**Published:** 2023-10-09

**Authors:** Jaquiele Jaciara Kegler, Eliane Tatsch Neves, Maria Ribeiro Lacerda, Diúlia Calegari de Oliveira

**Affiliations:** I Universidade Federal de Santa Maria. Santa Maria, Rio Grande do Sul, Brazil; II Universidade Federal do Paraná. Curitiba, Paraná, Brazil

**Keywords:** Nursing, Family, Concept Formation, Models, Nursing, Qualitative Research., Enfermería, Familia, Formación de Concepto, Modelos de Enfermería, Investigación Cualitativa., Enfermagem, Família, Formação de Conceito, Modelos de Enfermagem, Pesquisa Qualitativa.

## Abstract

**Objective:**

To communicate the experience of developing concepts for the construction of a care model through focus groups.

**Methods:**

An experience report on the development of concepts through remote focus groups with members of a research group from a public university in southern Brazil.

**Results:**

Focus groups were developed in which homogeneity and heterogeneity criteria were observed among participants. In addition to the concepts of the nursing metaparadigm, the concepts of care and family-centered care were developed, relevant to the nursing care model in question.

**Final considerations:**

Despite the challenges of conducting remote focus groups, they were suitable for the collective construction of concepts for a nursing care model, allowing the interaction of participants from different locations.

## RESUMO

**Objetivo:**

comunicar a experiência de elaboração de conceitos para a construção de um modelo de cuidado por meio de grupos de discussão.

**Métodos:**

relato de experiência sobre a elaboração de conceitos por meio de grupos de discussão remotos com integrantes de um grupo de pesquisa de uma universidade pública do sul do Brasil.

**Resultados:**

foram desenvolvidos grupos de discussão nos quais foram observados os critérios de homogeneidade e heterogeneidade dos participantes. Além dos conceitos do metaparadigma da enfermagem, foram elaborados os conceitos de cuidado e de cuidado centrado na família, relevantes para o modelo de cuidado de enfermagem em questão.

**Considerações finais:**

apesar dos desafios da realização da técnica de grupos de discussão por via remota, ela mostrou-se adequada para a construção coletiva de conceitos para um modelo de cuidado de enfermagem, possibilitando a interação de participantes de diversas localidades.

## RESUMEN

**Objetivo:**

comunicar la experiencia de elaboración de conceptos para la construcción de un modelo de cuidado a través de grupos de discusión.

**Métodos:**

relato de experiencia sobre la elaboración de conceptos a través de grupos de discusión remotos con integrantes de un grupo de investigación de una universidad pública del sur de Brasil.

**Resultados:**

se desarrollaron grupos de discusión en los que se observaron los criterios de homogeneidad y heterogeneidad de los participantes. Además de los conceptos del metaparadigma de la enfermería, se elaboraron los conceptos de cuidado y cuidado centrado en la familia, relevantes para el modelo de cuidado de enfermería en cuestión.

**Consideraciones finales:**

a pesar de los desafíos de la realización de la técnica de grupos de discusión de manera remota, se mostró adecuada para la construcción colectiva de conceptos para un modelo de cuidado de enfermería, permitiendo la interacción de participantes de diversas localidades.

## INTRODUCTION

Over the past five decades, Nursing has been recognized as an emerging profession, an academic discipline, and a science, mainly thanks to the great efforts of scientists, theorists, and scholars in the field. This recognition has only been possible due to the numerous discussions on the phenomena that characterize Nursing and the increased development and use of theories in research and practice^(1)^.

It is through theories that the processes of Nursing care are established, guiding the directions to be followed to implement theoretical assumptions in practice^(2)^. When the care process is conceptually and/or methodologically structured, it can be classified, depending on its degree of abstraction, as a care model, conceptual model, care methodology, among others^(3)^. There is still a lack of clarity about these terms among nursing scholars and professionals, sometimes resulting in their incorrect use^(2)^.

Care models are an important tool to guide Nursing practice. In addition to contributing to the development of differentiated and specific care, they promote knowledge and the discipline’s own development^(3)^. A care model is defined as a theoretical framework, consisting of the four concepts of the Nursing metaparadigm, as well as others that may be necessary, assumptions, and a care methodology. It must be translated through a diagram, that is, a graphic representation that seeks to reinforce the clarity of the model and the relationships between each of its elements^(4)^.

Some authors propose a structural hierarchy of Nursing knowledge, organized according to the level of abstraction, as follows: metaparadigm, philosophy, conceptual models, theories, and empirical indicators. The metaparadigm component is considered the highest level of abstraction, defined as the global concepts that identify the central phenomenon of interest in the discipline, the propositions that describe the concepts, and those that establish the relationships between them^(5)^.

Care theories and models are developed and improved through research, demonstrating their relevance to the development of Nursing knowledge. It is through research that their domain phenomena are identified, which can be defined through concepts^(6)^.

Concepts are terms that refer to phenomena that occur in nature or in thought, which can be abstract, such as feelings, or relatively concrete, such as temperature and pain. They are formulated in words, which allows the meanings attributed by people to realities in the world to be communicated and give meaning to phenomena that can be seen, heard, felt, smelled or touched directly or indirectly^(1)^. Thus, concepts, as an integral part of theories and care models, need to be constructed and/or improved through research. For this, discussion groups can be used as a data collection technique, enabling integration among participants and collective construction of concepts^(7)^.

As the problem of this study, there is a gap in scientific publications regarding the use of remote discussion groups. The use of remote data production/collection methods became more effective during the COVID-19 pandemic^(8)^. However, when consulting the scientific literature (LILACS) with the words “discussion groups” AND “remote” in January 2023, 13 productions were retrieved. However, none of them addressed discussion groups. This problem had already been identified during the study, which generated the need and also became the justification for reporting the developed experience.

## OBJECTIVE

To communicate the experience of elaborating concepts for the construction of a care model through discussion groups.

## METHODS

This is an experience report on the development of concepts related to family, newborns, nursing, care, neonatal intensive care unit, home, health-disease, and family-centered care for the construction of a nursing care model. This model aims to guide the practice of caring for families in neonatal intensive care units. This article is part of the thesis entitled “Family-centered nursing care model for neonatal intensive care - AMCORE”.

Participants were members of a child health research group at a public higher education institution in the state of Rio Grande do Sul, Brazil. The group, called Neonate, Child, Adolescent and Family Health - CRIANDO, was created in 2008 and is registered in the Directory of Research Groups of the National Council for Scientific and Technological Development (CNPq). As of October 2022, the group had seven researchers, six undergraduate students completing their final paper, three research fellows, five master’s students, and five doctoral students. The group has already graduated more than 20 master’s students, five doctoral students, and has published 175 articles, 34 book chapters, three books, and more than 200 papers in event proceedings.

To select participants, the inclusion criteria were: being a nurse or nursing undergraduate student and having at least two years of participation in the research group. Undergraduate students who had not completed the sixth semester of the nursing course were excluded. This criterion was adopted considering that students take the disciplines related to child and family care in the sixth semester of the course.

Firstly, a chart with the names of all the research group members was developed. Then, contact was made through a messaging app (WhatsApp), questioning each member about their participation time in the group and, for undergraduate students, the semester they were currently in. Subsequently, all members who met the inclusion criteria were invited by email.

For data collection, focus groups were utilized. The focus group is a technique through which the researcher seeks collective construction of ideas^(7)^. Two groups were organized to maintain a maximum quantity of up to 10 people per group on different days, considering the participants’ availability. The COVID-19 pandemic, declared by the World Health Organization in March 2020, brought numerous limitations to field research, requiring a restructuring by the researchers who had to resort to creativity. Interactive platforms were used to conduct the research during the pandemic, challenging researchers who needed to recreate their data collection methods^(9)^. Google Meet was the chosen application for remote meetings since it was already familiar to all participants, as it was also used in other remote activities of the research group.

An email was sent three days before the scheduled day for the focus group, containing information such as the purpose of the group, day, time, and access link, as well as the activity that should be carried out beforehand - each participant should choose three keywords for each concept (family, newborn, nursing, care, neonatal intensive care unit, home, health-disease, and family-centered care) that, in their opinion, represented them. The chosen keywords were presented by each participant on the scheduled day for each group.

After the concepts were developed, they were taken back to be discussed with members of the previous groups to validate them. Participants were contacted again, and they were asked to evaluate the concepts to judge if they were adequate or not. The concepts were sent in a Word file. Additionally, participants were requested to draw on an A4 sheet how they would represent the concepts in a diagram, and asked to send the drawing through the messaging application (WhatsApp).

Along with the email containing information about the focus group, a consent form was sent for participants to read beforehand. On the day of the groups, verbal consent was obtained from the participants regarding their agreement to participate and to have the meeting recorded in audio and video.

The discussion groups were coordinated by the researcher, author of the thesis project, and involved the participation of a research assistant who was a nursing undergraduate student and also a scholarship holder of the project. It is worth noting that this thesis is in the final stage of completion and has been approved by the research ethics committee.

## RESULTS

Two discussion groups (Group I and II) were developed for the elaboration of the concepts and two more (Group III and IV) for their validation. The groups were held on different days: Group I and II in December 2020 and Group III and IV in June 2021, all in the evening. Groups I and II were composed of seven research group members each, while Groups III and IV had five and six participants, respectively, from Groups I and II. Group I lasted one hour and eight minutes, Group II one hour and forty-three minutes, Group III one hour and fifteen minutes, and Group IV one hour and eleven minutes. Regarding academic level, six participants had a doctoral degree, three had a master’s degree, two were residents, and three were undergraduate students. The participation time in the research group ranged from 2 to 13 years.

In the composition of the groups, heterogeneity was sought, considering that different experiences and perspectives could enrich the discussions. Therefore, representatives of all academic levels - doctoral, master’s, and undergraduate - were included in both groups. Homogeneity was contemplated from the participants’ origin, who were part of a research group that addresses topics such as neonatology, care, and family, and were involved in the Nursing field, as professionals and/or students.

Groups I and II were operationalized as follows:

- Obtaining consent: Before recording, participants were asked if they agreed to participate in the research and if they authorized the audio and video recording of the group. It should be noted that consent was obtained from all participants;
- Motivation dynamics: The video “Lighthouse of Responsibility” was shown to participants as a way of thanking them for their participation in the research, highlighting the importance of each person in the construction of concepts;
- Presentation of participants: All participants introduced themselves to foster integration among them;
- Explanation of group dynamics: The objective of the group and how it would be organized were explained;
- Presentation and discussion of keywords: Each participant presented the three words they chose for each concept, explaining their choice. There was no specific order for the participants to present, allowing them to start the presentation at their own pace and continue as they saw fit. At times, there was overlapping speech; in these situations, one of them was asked to speak first and then the other;
- Summary of words: At the end of each concept’s discussion, the researcher summarized the participants’ keywords, which were compiled into a PowerPoint file by the research assistant. To maintain organization, presentation, discussion, and summarization were done by concept, following the order: newborn, neonatal intensive care unit, and so on.

The audio recordings from the discussion groups were transcribed into a Word document and analyzed using Braun and Clarke’s inductive thematic analysis^(10)^. This type of analysis is a method to identify, analyze, and report patterns (themes) in collected data, allowing for their organization and detailed description^(10)^. It was operationalized as follows:

- Data familiarization: exhaustive readings of the transcribed material were conducted and keywords for each concept were highlighted in the text, using eight different colors. Although the concepts were discussed separately for organizational purposes, it was possible to identify in the reading that they are interconnected, since words referring to one concept already appeared in the discussion of another. It is also noteworthy that all the keywords chosen by the participants were considered in the context of speech, that is, under what perspective they were being used, since the same word can have different meanings depending on the context;
- Initial code generation: all the highlighted keywords in the text were grouped in a table, exhaustively read, and categorized by color according to thematic affinity (Chart 1);
  **Chart 1:** Analysis process for the development of nursing care model concepts

Nursing	
Care Women and child healthcare Sensitivity Science Science in construction Assistance Health Proximity to the user Profession Attention Scientific knowledge Knowledge Professionalism Protagonism Vital category Sociocultural context Care management and administrative issues Proactivity Responsibility Empathy Challenge Donation Reference Link between team-family/child Promotion of health education	CARE - Care, care for women’s and children’s health, attention. SCIENCE - Science, science in construction, scientific knowledge, knowledge. REFERENCE - Proximity to the user, protagonism, vital category, reference, link between team-family/child. ASSISTANCE, MANAGEMENT, AND HEALTH EDUCATION - Assistance, health, care management and administrative issues, health education promotion.
- Theme generation: the keywords highlighted with the same color were grouped into themes (Chart 1). Sometimes it was necessary to return to the transcribed material to see the context in which the word was being used and thus identify which theme would be more appropriate to group it into;
- Review of themes: after the themes were generated, they were reviewed to confirm whether the keywords grouped in each theme adequately represented it (Chart 1);

Chart 1 presents an example of the analysis process that supported the development of each concept. On the left side of the table are all the keywords chosen by participants for the Nursing concept, while on the right side are the words grouped by theme.

- The generation of concepts occurred after the grouping of keywords by theme, and with the help of Portuguese language connectors and the theoretical reference of the thesis, concepts were constructed for family, newborn, neonatal intensive care unit, Nursing, care, home, health-disease, and family-centered care. It is noteworthy that the theoretical reference of the thesis was based on the publication entitled “Family-centered care for children with special health care needs” by Shelton, Jeppson, and Johnson (1987)^(11)^ and the concepts of dignity and respect, information sharing, participation, collaboration, family, and patient and family-centered care of the Institute for Patient-and Family-centered Care (2023)^(12)^.

The construction process involved several meetings among the research team, in which the concepts were restructured to become clear and to refer to the theoretical reference of the thesis. After construction, they were sent for Portuguese revision to analyze textual coherence and cohesion. The research team then met again to restructure the concepts based on the reviser’s suggestions.

After constructing the concepts, two discussion groups (Groups III and IV) were held for validation. The objective was for participants to validate the concepts constructed from the keywords discussed in Groups I and II and suggest any necessary changes. In these groups, participants presented their suggestions to others and reflected on which aspects of the concepts required adjustments. Modifications were suggested in both groups, and the research team discussed them, leading to their reformulation. Each participant also presented a diagram that they drew to represent the concepts.

At the end of Groups III and IV, participants were asked about their experience participating in the process of developing concepts through remote discussion groups. They evaluated the experience as very positive and of great learning, enabling the sharing of experiences. It was also considered a great responsibility since the concepts constructed will integrate a care model that will support Nursing practice. Furthermore, participants reported that despite remote groups still being something new, they were of great value as they brought together people from different locations. They considered it extremely important to validate the concepts and have the opportunity to suggest modifications to qualify them.

## DISCUSSION

The development of concepts through focus groups has proven to be challenging, especially when conducted remotely. Considering the difficulties related to the use of interactive platforms for data collection, such as reduced interaction between participants and the researcher, this may result in impaired construction of trust and proximity necessary for data collection^(9)^. In this study, most participants were already familiar with each other due to their participation in the research group, which facilitated open discussions from the outset.

Focus groups emphasize the collective construction of ideas^(7)^, which was observed in this study where participants actively collaborated in the development of concepts, initially with the keywords they chose and later with their validation. Considering that focus groups aim to obtain data that allows for the analysis of participants’ contexts and worldviews^(7)^, heterogeneous groups were formed with regard to their experiences related to the theme and academic level. This facilitated richer discussions and exchange of knowledge and experiences, which participants also noted as positive.

In conducting the group, we sought to assume the role of fostering reflections and discussions, directing questions to the group as a whole, as recommended by the focus group technique. Conducting a remote group is fraught with challenges, as Internet connection issues often necessitated repeating questions or overlapping speech. During the group, the researcher should establish reciprocal contact with the participants, initiate the discussion with a broad question, stimulate participation and interaction, allow the organization to be led by the group itself, formulate questions that generate narratives rather than mere descriptions of facts, direct questions always to the group, and intervene only when necessary^(7)^.

In the analysis of the discussion groups, the inductive theme proved to be suitable for the elaboration of concepts, considering that it allowed for the identification, analysis, and reporting of themes in the collected data that, when grouped, supported the construction of concepts. The inductive theme advocates for an analysis that is guided by data rather than a pre-existing coding framework^(10)^. An important aspect of the analysis was respecting the context of the participants’ speech, as, when working with keywords, depending on the context in which they were used, they could assume various meanings. This type of analysis recommends that data extracts be coded more broadly, retaining some of the surrounding data so that the context is not lost^(10)^.

As previously mentioned, the concepts constructed from the analysis were care, family-centered care, nursing, family, newborn, neonatal intensive care unit, home, and health-disease. In this study, the human being metaparadigm is represented by the concepts of family and newborn, the environment metaparadigm by the concepts of neonatal intensive care unit and home, and the health metaparadigm by the concept of health-disease. The purpose of a metaparadigm is to synthesize the intellectual and social missions of a discipline and set limits on its phenomena of interest. In this sense, detailing the concepts and propositions that represent a discipline’s subject matter allows its members to share their greatest interests with members of other disciplines and the general public^(5)^. In addition to the nursing metaparadigm concepts, the concepts of care and family-centered care were developed, considering their relevance to the construction of a family-centered nursing care model for neonatal intensive care.

The diagrams presented by the participants allowed for the visualization of the relationship between concepts based on their perceptions and could serve as inspiration for the diagramming of the nursing care model. Diagrams are important strategies when seeking to schematize the relationship between concepts^(13)^ and are also an important element for understanding nursing models.

### Study Limitations

One limitation of the study is that it is a narrow focus only in the area of neonatology, making it necessary to expand the methodology to other areas within the field of nursing.

### Contributions to the Nursing Field

It is believed that this report will contribute to the dissemination of the technique and encourage researchers who, faced with difficulties, may use creativity to collect data for their research. Additionally, it presents possible strategies in the development of care models, which are essential for the construction and advancement of the science of nursing.

## FINAL CONSIDERATIONS

In this study, the technique of focus groups proved to be adequate for the collective construction of concepts for a nursing care model. Despite the challenge imposed by the COVID-19 pandemic, the use of an interactive platform facilitated data collection, allowing participants from different locations to come together. Moreover, participants considered it extremely important to validate their concepts and suggest modifications to improve them. The heterogeneity of the groups was also positive, as it provided richer discussions and exchange of knowledge and experiences. Regarding the analysis, the inductive thematic approach proved to be suitable, as it enabled the identification of themes from the transcriptions of the discussions in the groups, which supported the formation of the concepts.

It is suggested to explore the use of remote means for group data collection, as it proved to be an excellent tool for interaction, overcoming the difficulties arising from social distancing among participants. It allowed for the heterogeneity of the group, enriching the group discussion, a primary characteristic of group techniques for producing data in qualitative research.
